# Understanding patients’ experience living with diabetes type 2 and effective disease management: a qualitative study following a mobile health intervention in Bangladesh

**DOI:** 10.1186/s12913-019-4811-9

**Published:** 2020-01-09

**Authors:** F. Yasmin, L. Ali, B. Banu, F. B. Rasul, R. Sauerborn, A. Souares

**Affiliations:** 1 Heidelberg Institute of Global Health, Heidelberg University Hospital, 69120 Heidelberg, Germany; 2 Faculty of Health/Department of Human Medicine, University Witten/Herdecke, 58448 Witten, Germany; 3 Bangladesh University of Health Sciences (BUHS), Mirpur-1, Dhaka, 1216 Bangladesh; 4 BRAC James P. Grant School of Public Health, BRAC University, Mohakhali, Dhaka, 1212 Bangladesh

**Keywords:** Mobile health, Interactive voice call, Call center, Type 2 diabetes, In-depth interview, Bangladesh

## Abstract

**Background:**

In 2017, 425 million adults worldwide had diabetes; 80% were living in low and middle-income countries. Bangladesh had 6.9 million adults with diabetes; death from diabetes comprised 3% of the country’s total mortality. This study looked at different factors (personal, familial, social, and financial) affecting both the life of patients with diabetes type 2 and the management of the disease. It also explored patient’s perception of the mobile health intervention in the context of disease management and helped to explain the findings obtained from the quantitative part of this study.

**Method:**

The study was a mixed-method, sequential explanatory design. A mobile health project (interactive voice call and call center) was implemented in Dhaka district, Bangladesh from January to December, 2014. Patients received treatment at the outpatient department of Bangladesh Institute of Health Science Hospital, Dhaka, Bangladesh, were included in intervention and control groups of the main study following a Randomized Control Trial. Among them, a total of 18 patients (9 + 9) were selected purposefully for the qualitative study, which was conducted in July, 2015. The sample was selected purposefully considering the age, sex, socio-economic status and proximity of living due to the political instability of the country during the data collection period. The interviews were transcribed and analyzed applying investigator triangulation.

**Results:**

Most patients stated that diabetes has affected their lives. In general, both groups´ evaluation of mobile health services were good and both regarded the recommendations for medication, diet, physical exercise, and other lifestyle behaviors (use of tobacco and betel nuts) as helpful. The cost of overall treatment (medications, physician consultations, laboratory investigations), the lack of availability of safe public places for physical exercise and unfavorable weather conditions (heat, rainfall) were mentioned as barriers to the overall management of the disease.

**Conclusion:**

A patient-centered mobile health intervention supported by a collaborative patient-provider relationship, a strong family support system, available public spaces for exercise and the introduction of a functional public health insurance system could be beneficial for the better management of diabetes.

## Background

Non-communicable diseases (NCDs) have emerged as a serious challenge for both health and economic development [1]. NCDs have considerably increased premature mortality and morbidity, and put a double burden of diseases (already existing communicable and newly added non-communicable) onto the health system through increased service utilization and overall treatment cost [1, 2]. This burdening of the health system hinders both poverty alleviation and sustainable development of low and middle-income countries (LMICs) [2]. Diabetes is an increasing public health concern for both developed and developing countries. In 2017, 425 million adults worldwide had diabetes, with a prevalence rate of 8.8%. Among them 80% were living in LMICs. Bangladesh had 6.9 million adults with diabetes with an age adjusted prevalence rate of 8.4% [2]. The prevalence is higher in urban than rural areas [3]. Diabetes related deaths comprised 3% of the country’s total mortality [4]. The annual cost of diabetes mellitus (DM) is USD 51.4 per person in Bangladesh [2]. In 2011, up to 40% of people with DM could not afford treatment [5]; the high percentage (67%) of out-of-pocket payment (OOP) is one of the main reasons [6]. Access to health care service is another major problem of health system of Bangladesh. Bangladesh is experiencing a demographic and epidemiological transition. With a growing elderly population the modification of lifestyles linked to rapid urbanization (3.5% annual urban growth) leads to increased sedentary lifestyles, higher calorie consumption, and more stressful life conditions. All these elements, combined with a lack of awareness about healthy lifestyles contribute to the increasing prevalence of diabetes type 2 (DM 2) and other NCDs [7, 8].

Adherence is defined as the extent to which a patient’s behavior corresponds with the agreed medications and lifestyle recommendations from a health care provider [9]. Non-adherence causes higher mortality and morbidity, the development of complications, poor outcomes of the disease, and an overall low health related quality of life (HRQoL) [10]. Non-adherence also has some economic consequences including repeated physician visits and medical investigations, increased hospitalization, disability, and premature death [11]. Adherence is considered more important in cases of chronic disease conditions like DM 2 as adherence to any treatment regimen is mostly inversely proportional to the duration of the treatment; it is usually higher among the patients with acute conditions compared to the patients with chronic conditions [12]. This challenging situation raised the question of how to ensure the sustainable and cost-effective treatment of DM 2 that will not challenge further the country’s health system and economy. The first measure is the early diagnosis and treatment with medications and the adoption of healthy lifestyles, particularly better dietary practices and physical activities to delay the development of complications and prevent premature mortality. As a potential solution, the use of mobile health (m-Health) came into consideration as an addition to the existing health care system to increase patients’ adherence and thereby, improve disease outcomes.

In Bangladesh, 97.5% (156 million people out of 160 million) of individuals and 89% of households have mobile phone coverage [13, 14]. This wide range of coverage offers the opportunity to use mobile phone services (such as SMS- short message services, voice calls, call center etc.) to reach people to provide more personalized health care. This could potentially enhance adherence and disease outcomes, which in turn may help to reduce the health system burden and health care cost [15]. But the utilization of m-Health services has not yet reached its full potential. This is especially the case for the following sub-groups: the elderly, who lack both information on modern technology and motivation to use it (factor age); females, who have less ownership as a result of male control over household mobile phone ownership (factor sex); illiterate people, who have less access, information, use, acceptance and understanding of modern technology (factor literacy); low income groups, who lack money to top-up mobile phone (factor economic status); and the population living in rural and hard-to-reach areas, who lack to a mobile network, to infrastructure and to electricity (factor geographical location) [16–25]. Moreover, the patient’s willingness to use such intervention as an additional treatment provision and the factors related to the use of and to the patient adherence is also yet not evident in the resource-limited settings of Bangladesh. Considering this need, an m-Health project was implemented in the capital city Dhaka, Bangladesh for one year within the scope of the study.

## Methodology

### Study objectives and design

A mixed-method, sequential explanatory design was used in this study. The quantitative Randomized Control Trial (RCT) survey was followed by a qualitative component. Quantitative data were collected and primarily analyzed at first. The results of the quantitative part were used to develop the qualitative data collection tool. Collection and analyses of qualitative data took place in sequence to help explain, and/or elaborate the quantitative results obtained, which was one of the objectives of the study. The other objectives of the qualitative component were to explore the patient’s perception of using m-Health services as an added provision in the treatment of DM 2, and to ascertain the influence of potential contributing factors (e.g. personal, familial, social, financial, political) on patient adherence. Patients were considered to be adherent (self-reported) when following the recommendations of their physicians regarding medication, diet, physical exercise, use of tobacco and betel nuts. The m-Health project was implemented for one year (January to December, 2014) in Dhaka, Bangladesh. The project was a joint collaborative study by Heidelberg Institute of Global Health (HIGH), Heidelberg University Hospital, Heidelberg, Germany and the Bangladesh University of Health Sciences (BUHS), Dhaka, Bangladesh.

### Services offered under the m-Health project

There were two types of services offered under this project to the patients who were enrolled (intervention group). First, patients were enrolled in a reminding system (through interactive voice call) to follow their recommendations for medication, diet, physical exercise, hospital visits, and other lifestyle modification measures. The project had detailed socio-demographic and economic information about the patients including their age, sex, education, work and financial status and family history. Furthermore, information about the disease and treatment were also included: stage, related complications (if any), blood glucose control level, prescribed medicine/s (name, dose, frequency, oral or insulin), dietary advice (according to Bangladesh Diabetes Association guide book-BADAS), physical exercise advice, and advice on avoiding smoke or smokeless tobacco and betel nuts. The voice call interaction was personalized and considered all the above mentioned information collected during the baseline survey. Patients received one call over their mobile phone every ten days, except Fridays and other national holidays. In cases where a patient did not answer the call, it was repeated three times on the same day at one-hour intervals. In other cases, if it was not convenient for a patient to talk, the call was repeated at a convenient time on the same day. If a patient was still not reachable, s/he was called again within the next three days. A call was considered successful if a full conversation with the patient was made. The average duration of each call was ten minutes. Communication was in Bangla, the local language. The research assistant in charge of the voice calls maintained a register and recorded all communications with the patients. In addition, patients received a reminder call one or two days prior to their scheduled hospital visits. The patients enrolled under the m-Health project could also receive services from the 24/7 call center. All health-related suggestions, consultation and treatment, and/or referral advices were provided by the physicians from the call center. Interactive voice calls and the call center were used as the implementation tools to better reach patients irrespective of their age (including elderly), education (the literacy rate in Bangladesh is only 62.5%), or socio-economic status (SES) [14]. Possession of a personal mobile phone was considered as one of the inclusion criteria for patient enrolment, though no patient lacked a personal mobile phone during enrolment. The BIHS hospital was responsible for patient reminder services and the Telemedicine Reference Center Ltd. (TRCL) & e-Health Solutions for the call center services. The services were provided free of cost during the intervention period.

### Sampling procedure

The patients receiving treatment for DM 2 at BIHS hospital were included in the main survey (quantitative) following a RCT design. A total of 320 patients (160 in the intervention and 160 in the control groups) were included in the main survey. Among them, a strategic purposive sampling was applied to select the interviewees from the intervention and the control group for the qualitative survey [26]. Because of the unstable and violent political condition of the country during the data collection period, proximity to the hospital was the primary criteria to select the patients. In addition, patients’ age, sex, and SES (education, occupation) were also considered. The interviews were conducted till the saturation point was reached [26]. At the end, an equal number of in-depth interviews were conducted from both groups, covering a total of 18 (9 + 9) patients. The reason for including patients from the intervention and the control group was that the patients from the intervention and the control groups were expected to have a different experience of managing their disease due to the continuous monitoring and support they were getting through the m-Health project.

### Pre-testing and conduction of interviews

A semi-structured interview guide was developed for this study (additional files 1 & 2). Four trial interviews (two from each group) were conducted and necessary corrections and adaptions were made prior to the main data collection phase. These four trial interviews were not included in the main analysis. The study objectives were explained to each participant before proceeding for the interview, and a written informed consent was obtained (additional file 3). The patients of the control group were told about the m-Health intervention before proceeding for the part “perception of m-Health”. All interviews were recorded with the permission of the patients. Interviews took place at BIHS hospital in July, 2015. Bangla was used to conduct the interviews. Each interview lasted for about 30–40 min.

### Data transcription and analysis

One study assistant transcribed the recorded version of the interview while another translated the Bangla transcription into English. The entire transcription was strictly monitored, and a quality check of the transcription and translation was performed during and after the completion of the transcription by a member of BUHS. The final check was done by the first author before starting the analysis. A deductive approach was used since the initial codes were guided by the conceptual framework of the study (theory) (Table 1) and the relevant quantitative study results [27]. Reading the entire transcripts was the first step of the analysis, which helped to identify the evolving themes and to connect the theory to the context. The code structure was developed following a hybrid approach. A first coding frame was developed based on the literature (deductive) and additional themes were added based on the material collected (inductive). The coding frame was first developed by the first author, and then revised and finalized after discussions with the last author. The final step of the analysis was connecting and inter-relating themes while constructing a narration. Data analysis was done using QSR NVivo 10 software.

**Table 1:** Conceptual Framework of the Study (factors of patient adherence in long term therapies)

Socio-economic and political factors	
- Social factor: norms, regulation, social network, support	
- Political and social unrest	
Health system related factors	
- Health policy and strategy	
- Health facility/institution	
- Financial support (user fee, insurance)	
- Medication supplies	
- Accessibility	
- Affordability (cost)	
- Availability	
- Quality of care	
Patient related factors	
- Socio-economic status: age, sex, marital status, SES	
- Literacy	
- Employment	
- Living conditions	
- Transportation means	
- Psychological aspects: locus of control, self-efficacy	
- Knowledge, believes, perception, practices about diseases and illnesses	
- Motivation and attitude towards therapy	
- Expectation of the patient	
- Peer pressure	
- Stress, depression	
- Patient readiness to change, accept diagnosis, and treatment regimen	
Provider related factors	
- Provider satisfaction	
- Lack of financial and other incentives	
Patient-provider relationship factors	
- Information exchanged regarding diseases and treatment	
- Health education	
- Patient/provider satisfaction	
- Patient-provider relationship	
- Communication	
- Trust	
Therapy related factors	
- Complexity of treatment	
- Route of administration	
- Duration of the treatment	
- Failure of previous treatment	
- Diabetic medication (oral, insulin, and/or combined)	
- Cost	
- Medication other than anti-diabetic	
- Side effects of medication	
- Frequent medication change	
- Previous treatment failure	
- Degree of behavioral change required	
Disease related factors	
- Duration	
- Stage	
- Complication	
- Presence of comorbid condition	

## Results

The results of the study are organized into seven sections: the perception of m-Health, life with diabetes, management of hospital visits and hospital services, management of medication intake, dietary practice, physical exercise, and political situation of the country during the study. The findings are illustrated using the quotations from the interviewees. The characteristics of the study participants are presented in the Table 2.

**Table 2:** Socio-demographic characteristics of the study participants

Patient ID	Sex	Age (in range)	Educational status	Marital status	Work status	Family income in Euro (Monthly)	Duration of disease (in years)
Intervention 1	F	50–59	SSC	Married	Housewife	234–465	10
Intervention 2	M	60–69	PSC	Married	Small business	35–116	15
Intervention 3	F	50–59	GLC	Married	Housewife	> 465	5
Intervention 4	F	40–49	SSC	Married	Housewife	35–116	6
Intervention 5	F	40–49	SSC	Married	Housewife	> 465	12
Intervention 6	M	40–49	SSC	Married	Small business	234–465	5
Intervention 7	F	50–59	SSC	Window	Housewife	> 465	7
Intervention 8	F	40–49	SSC	Window	Housewife	> 465	12
Intervention 9	F	50–59	SSC	Married	Housewife	> 465	5
Control 1	F	70–79	SSC	Window	Housewife	Don’t know	5
Control 2	F	40–49	SSC	Married	Private company	> 465	7
Control 3	F	50–59	SSC	Window	Housewife	234–465	10
Control 4	F	50–59	SSC	Married	Housewife	117–233	4
Control 5	F	30–39	NFS	Married	Small business	35–116	7
Control 6	M	40–49	SSC	Married	Small business	35–116	2
Control 7	F	50–59	SSC	Married	Housewife	117–233	15
Control 8	M	60–69	PGC	Wife died	Retired person	117–233	15
Control 9	F	30–39	SSC	Married	Housewife	117–233	4

NFS = No formal schooling, PSC=Primary school completed, SSC = Secondary school completed, GLC = Graduation level completed, PGC = Post-graduation level completed

### Perception of m-health project

Before asking about the perception of the m-Health intervention, patients of the control group were familiarized with the intervention and then asked to share their views about m-Health with respect to the management of DM 2. In response to the question, patients from both groups mentioned it would give them a very good feeling in general if someone took (eight from the intervention group) or if someone would take (five from the control group) care of them and enquire about their health issues. For example, “*I feel good as she* [research assistant] enquires *about me... It always gives you a good feeling if someone cares about you*” (Intervention 4: female, SSC, housewife). Two-thirds of the patients said that receiving a call served (nine from the intervention group) or would serve (three from the control group) as a good reminder and control over their health and disease conditions. It made (intervention group) or would make (control group) them aware to follow the health care provider’s advice and change their practices accordingly. For example, “*I become automatically conscious, when she* [research assistant] *calls me. I figure it out whether I am doing right or not. I noticed that when she calls me and asks me about my disease, it helps me to become conscious about my disease and stay well*” (Intervention 2: male, PSC, small business). And “*When she* [research assistant] *calls me, and … Hello uncle, how are you? Even if she says up to this, that’s already a control, a good control* [for me]” (Intervention 6: male, SSC, small business). Patients from the intervention group reported that they discussed with the research assistant about their other physical problems or diseases as well and received suggestions. The research assistant reported getting calls back from several intervention group patients during the implementation period that expressed their gratitude and showed their appreciation of the links being created by the m-Health project. In contrast, only one patient from the control group expressed her concern that repeated calling might be irritating for some patients.

Almost all patients from both groups showed their interest in the availability of a 24/7 call center service. They described it as beneficial in general, particularly in case of an emergency (especially at night). But none of the patients from the intervention group called during the intervention period, since they did not have any emergencies and had regular hospital visits. The intervention was free of cost during the implementation period. In response to the question whether patients would like to pay for such services in future, more than half of the patients (seven from the intervention and four from the control group) said that they would pay. The suggested amount varied from USD 3.00 to 7.50 per month depending on the financial capacity of the patients. Some of the female patients (three from each group) stated the need to discuss or get permission from their family members (husband or son), since they were not the earning members of the family. One patient from the control group mentioned her financial inability to pay, but regarded the service as a good initiative. One patient from the intervention group stated that the information and the advice provided by the project were already known, and therefore, she was not willing to pay. The patients provided several suggestions for further improvement of the services: (1) organize monthly discussion sessions to give the patients the opportunity to talk, share, and discuss about their health conditions and problems, (2) provide advices and inform them about the use and side-effects of traditional medicines, (3) inform them about other health-related issues, and (4) offer free physician consultations and laboratory tests as part of the m-Health service provision.

### Life with diabetes

The patients were almost equally divided into three subgroups: fully satisfied, moderately satisfied and not satisfied at all regarding their life with diabetes. Most of the patients (16 out of 18) expressed their life as “not like before” or “different” or “changed life style”. They expressed a tense (four from the intervention group), irritated or restless (three from the control group), or helpless feeling (one from each group) and an upset feeling with a life taking medications (one from the intervention group). Some patients mentioned that life with diabetes means an adjusted or restricted life resulting from the demands of the management of the disease, which forced (in reality or in perception) the patients to limit their lifestyle (e.g. life-long medication intake, dietary restrictions, regular physical exercises, check-ups, laboratory tests and self-care), the development of complications and lifelong disabilities, and/or changes in their family and social life (four patients from each group). But on the other hand, few patients expressed such routine life as a positive way to maintain their good health. More than three quarters of the patients from both groups stated that they have other diseases associated with diabetes. The most commonly mentioned diseases or health conditions were heart diseases, high blood pressure, joint pain (arthritis), kidney problems, and physical discomforts such as general weakness, pain all over the body, and dry throat.

Almost all patients from both groups mentioned that they faced no changes or problems in their personal or family life due to having diabetes. They added that awareness and mutual support already existed in their families, as some of the family members also have diabetes (partner, parents or siblings). None of the patients faced any difficulty in their social life because of having diabetes. Patients also mentioned that no stigma or social pressure existed anymore since almost all families have diabetic patients now-a-days. More than half of the patients (seven from the intervention and four from the control group) irrespective of their economic status mentioned that they had an extra financial burden at a personal or family level because of the cost of the treatment. In contrast, some patients, irrespective of their economic status, mentioned explicitly that spending the extra money for treatment was not a problem for them. Patients with a perceived financial problem or burden reported lesser satisfaction with life than those without a perceived financial problem or burden. Quotes related to this section are presented in Table 3.

**Table 3:** Life with diabetes- quotes from the patients

Satisfaction with life	“*I have no sorrow as I am not the only one; there are colleagues around me who have diabetes and are still nicely getting around with their work and family. There is no effect of diabetes on my life … It’s a normal thing now*” (Control 2: female, SSC, private company).
	“*I get tension when my* [blood] *pressure or diabetes [blood glucose] increases. Now I have to live on medications, still I manage as long as I have money ... I am not fully happy, again not fully unhappy*”. (Intervention 2: male, PHC, small business).
Different/changed life style	“*Living this life means living with medications*” (Control 1: female, SSC, housewife).
	“*There are obviously some changes in my life because of my physical illness which makes me feel helpless*” (Intervention 8: female, SSC, housewife).
	“*After maintaining medication, diet, and other routines* [e.g. regular exercise]*, I feel that it is not that much of a problem these days*” (Intervention 8: female, SSC, housewife).
Family issues	“*There is no problem at my family. We have eight to nine members in my family including my parents and siblings who have diabetes*” (Control 4: female, SSC, housewife).
	“*I don’t face that much problem in my family life as both of us* [husband and wife] *have diabetes*” (Intervention 2: male, PSC, small business).
Social issues	“*Diabetes is now in all houses. So, everyone knows that it is a normal disease. So it is no more a social problem*” (Intervention 3: female, GLC, housewife).
Economic issues	“*It is an extra burden, definitely it is an extra financial burden*” (Intervention 4: female, SSC, housewife).

### Management of hospital visits and hospital services

Regular hospital visits were reported by three-quarters of the patients (seven from the intervention and six from the control group). Self-motivation and family support were the reason for regular hospital visits. Most of the patients (seven from the intervention and eight the from control group) visited the hospital alone since they believed that it was their own responsibility. Few patients said that their husband or wife accompanied them during their hospital visits (two from the intervention and one from the control group). Financial difficulties were mentioned as the main reason for irregular or delayed hospital visits. For example, “*I have to arrange money to come to the hospital. Coming to the hospital always costs money*” (Control 9: female, SSC, housewife). Lack of self-discipline and family problems such as the need to take care of children and responsibilities at work and at home were also mentioned as the cause of delayed hospital visits. However, none of the patients reported to fully omit the hospital visits.

Regarding the hospital services, less than half of the patients (two from the intervention and five from the control group) expressed their full satisfaction. Patients mainly (six patients from each group) expressed their satisfaction with the respectful behavior of the physicians and their treatments and suggestions. But some patients complained about the lack of attention and consultation time provided by the physicians. Patients also cited about long waiting times, the unfriendly behavior of hospital staff at the registration desk (due to shortage of manpower) and the higher cost of the hospital services.

### Management of medication intake

Less than one third of the patients (two from the intervention and three from the control group) were self-motivated to take their medications as advised by the physicians. All patients mentioned that family support is very important in ensuring regular medication intake practice. But only less than half of the patients (five from the intervention and three from the control group) reported getting both emotional (encouragement and reminders to take medications, mutual support from the spouse) and financial support from their family. Mutual support was also stated in this regard. On the other hand, more than half of the patients (five from the intervention and six from the control group) mentioned not having any family support. Financial problems were mentioned as the main reason for omitting medications (two from the intervention and three from the control group). In addition, forgetfulness, laziness, anger and irritation related to the need to take medications were also mentioned as the reasons for irregular medication intake practice. Ramadan was also stated as a reason for altered medication intake practice. Quotes related to this section are presented in Table 4.

**Table 4:** Management of medication intake – quotes from the patients

Self-motivation	“*I have to take medications, because I have to live and stay healthy. So, I take medications regularly*” (Intervention 1: female, SSC, housewife).
	“*No matter what happens, I give my treatment the first priority*” (Control 3: female, SSC, housewife).
Familial support (emotional and financial)	“*I try to make her* [wife] *aware and she* [wife] *does as well. We* [husband and wife] *do mutually take care of each other as both of us have the same disease* [diabetes] *and stress*” (Intervention 2: male, PSC, small business).
	“*My husband is very conscious about my medications. He gives me money to buy the monthly medicines at a time. So, there is not even a single day interruption of my medication intake*” (Control 4: female, SSC, housewife).
	“*My husband even doesn’t know if I have diabetes or any other diseases. He even never asks me what medications I am taking*” (Control 2: female, SSC, private company).
Financial constraint	“*Normally, I do not miss insulin. But sometimes I cannot take it* [insulin] *due to money. If I don’t have money, simply I cannot buy insulin*” (Control 5: female, NFS, small business).
Anger as a barrier to adherence	*“Sometimes I skipped medicines due to anger. I feel angry with myself why I have to take medicines all the time*” (Intervention 6: male, SSC, small business).

### Management of dietary practice

More than two-thirds of the patients (eight from the intervention and seven from the control group) reported following their dietary recommendations. Self-motivation and positive family support were reported important in maintaining adherence. Most patients said that they shared the same food with their family members, but at a lower quantity. More than half of the patients (seven from the intervention and three from the control group) said that their family members made them aware about their suggested food items and helped them to avoid prohibited food items. Others (two from the intervention and one from the control group) reported that their family members changed their food intake practice to match with their requirements. For example, “*My husband doesn’t eat many things because of me. As I cannot eat sweets, he also does avoid it. He says that if you cannot eat, how can I eat?*” (Intervention 1: female, SSC, housewife). And “*They* [family members] *bring* [only] *those particular food items from the market which I am advised or allowed to eat*” (Intervention 3: female, GLC, housewife). On the other hand, some patients (three from the intervention and five from the control group) reported having no support from their family members. Few patients talked about their irregular dietary practices. The reasons for such irregularities were a lack of time (work pressure at home or at work place) and a lack of self-motivation (laziness, anger due to having food restrictions, and a desire to eat seasonal fruits), other illnesses, and extended family visits. Ramadan as a special circumstance of changed food intake practice in pattern, frequency and timing was also mentioned.

### Management of physical exercise

More than half of the patients (four from the intervention and six from the control group) engaged in physical exercise and walked regularly for 15 to 60 min on an average per day. Patients regarded physical exercise as a good way of keeping diabetes at a controlled level. For example, “*Walking is the main medication for diabetes*” (Intervention 3: female, GLC, housewife). Most patients reported that family members motivated and inspired them (seven from the intervention and six from the control group) and in a few cases even their husband and/or son walked with them (two from the intervention group) regularly. Social connection was also mentioned as an encouraging factor for this. For example, “*I feel encouraged to walk as I made friends there* [during walk]*. I meet everyone while walking and we talk about various topics including our diabetes. I came to know many things from others during walking*” (Control 7: female, SSC, housewife).

Patients mentioned physical problems such as weakness, palpitation, and pain (leg and chest) as the main reasons for irregular physical exercise (four from the intervention and one from the control group). Lack of time due to work pressure at home or at work place as well as unfavorable weather conditions such as heavy rainfall, extreme heat, and humidity were also mentioned. For example, *“In monsoon season, sometimes I cannot walk continuously for 4-5 days due to rain”* (Intervention 8: female, SSC, housewife). Unsafe and uneven pedestrian paths or a lack of safe public places (e.g. park) to walk were also mentioned as a barrier to regular walking. Several patients stated that they walked at home (in the corridor or on the terrace) because of their physical problems and/or unfavorable outside conditions). Ramadan was also cited as a factor for irregular physical exercise practice due to the potential risk of a hypoglycemic attack and general weakness resulting from prolonged fasting. But most of the patients reported that they continued their physical exercise (walking), mainly in the morning, in order to avoid hypoglycemia in the evening while fasting.

### Political environment of the country

All patients stated that the unstable political condition of the country during the data collection period was problematic. Despite this, most of the patients (seven from each group) reported that they were able to visit the hospital regularly because their home was near to the hospital. One patient showed her strong determination even under violent condition. “*Medication stock is finished; I have to buy my medications even if no rickshaw* [local transport] *is available on the road. I have to walk to buy my medications. During that time* [the period of political instability]*, there was definitely some insecurity on the road and I was worried. But still I had to do everything* [needed for my treatment]” (Intervention 3: female, GLC, housewife). Only a few patients mentioned that they omitted hospital visits due to the possible violence that might happen on the way to the hospital. Most of the patients stated that they did not suffer financially from the political instability.

## Limitations

The study has several limitations. First, the patients of the study were recruited from a tertiary care hospital (BIHS) in Dhaka, Bangladesh. Therefore, the patients were unlikely to represent all patients with DM 2 in the country, which influences the transferability of the results. Second, female participation was significantly higher in both groups, which was found consistent with the hospital records. There was a conscious intention to increase the male participation in the interviews to enhance the male perspectives in the study. Nevertheless, the study provided an opportunity to get a deeper understanding of the experiences of the patients living with DM2 and their difficulties of being adherent to medication, diet, exercise, and other lifestyle recommendations. In addition, it gave a detailed understanding of the perception of m-Health intervention in disease management.

## Discussion

In this study patients from both groups regarded the ability of m-Health services to support managing their disease as good. The results showed that the m-Health calling service was helpful and made the patients pay more attention to their own health and to stay adherent, particularly to dietary advice and hospital visits. Cost was reported as the decisive factor linked to non-adherence to medication intake and physical problems or co-morbidities were reported as the main difficulties in patients following physical exercise recommendations. The study showed the importance of the personal relationship developed between the patient and the person in charge of calling which made the patient feel responsible for taking care of their own health. Considering the low literacy rate in Bangladesh (only 62.5%) and the comparatively older age group of the patients included in our study, voice call interaction in local language was used as the tool in study [14]. The assumption of this study was that the older and less-educated patients had a lower awareness and acceptance of modern technologies used in m-Health interventions, which was confirmed by another study conducted in the USA [28]. In this study, patients’ concerns were addressed through an option of two-way voice call communication with the research assistant. The personally tailored communication provided the patients with a feeling of being specially taken care of, which may have increased their motivation and responsibility to follow their treatment advices in this study. One review article and two more interventional studies conducted in the USA and Malawi stated that voice call was preferred by the patients due to the personalized information received [29–31]. Other studies also reported that the tools work well when it is customized, and when the language, and content of the tools are highly relevant to the individual patient [30, 32]. Patients appreciated and expressed their interest in using a 24/7 call center in this study, though no patient called during the intervention period since none of them had an emergency and almost all had regular hospital visits. WHO reported that the health call centers and emergency phone services are the most frequently used m-Health initiatives globally [33]. In this study, the results may have occurred because the interviewed patients were living near to the hospital and still preferred face-to-face consultation. Nevertheless cost was mentioned by the patients to be the decisive factor for hospital visits. M-Health services could help in this regard by reducing the cost of face-to-face consultation, removing transportation cost, and cutting the indirect cost of time off from work either due to coming to or waiting at the health facility, both for patient and attendant. But it does not remove fully the financial barrier since the cost of using a mobile phone is still high for most of the people in Bangladesh [15]. At the provider level, m-Health could help to remove the additional load on already burdened health facilities through unnecessary visits [34]. In this study, most of the patients showed their interest in getting m-Health services in the future even if they have to pay since they found the services beneficial to them. Despite their interest, some patients could not confirm their future enrollment because of financial problems or because they were not empowered to take any financial decision. Most of the interviewed patients in this study were housewives. Another study conducted in Bangladesh found that generally women (housewives) have less say in the financial decision-making process than men [25]. A subsidization of the m-Health service fees could be implemented to reduce the financial burden on the household level [35]. A study conducted in Bangladesh evidenced a similar level of adherence among patients who received advices from face-to-face consultations and from m-Health services [36]. Therefore, considering the different challenges of the health system in Bangladesh as well as in other LMICs and the positive results of m-Health interventions; it could be used as a complementary option to the main service provision. A positive experience already took place in Bangladesh: the Grameen Health line. The program provided medical advices to approximately 10,000 callers per day in 2007 [35]. A public-private partnership (PPP) with other non-government organizations (NGOs) and telecommunications companies could be introduced to provide free or subsidized mobile health care services to the population, especially to those who need the accessibility, availability and affordability of the services for better treatment outcomes.

Most patients during the interviews stated that their lives were different because of the need to take medications on time, dietary restrictions, daily exercise routines, and regular hospital visits. Patients also reported about physical problems and emotional stresses such as tension, depression, frustration, anger or irritation due to living a life with diabetes. These findings were confirmed by other studies in these areas [37, 38].

Most of the patients in this study said that they received positive social support (from family members, friends and other members of the society) and did not suffer from any stigma related to their disease. According to other studies, social support helps patients to manage diabetes-induced stress and depression and motivates them in their self-care activities; family members are considered to be the most significant source of social support for self-care activities in chronic disease conditions [39–41] Other studies also showed that social and family support promoted adherence by inspiring positivity and self-confidence and by encouraging patients’ ability to cope and to take control over their disease [40]. This study revealed that family support through reminders and financial support resulted in more regular hospital visits; this finding is similar to another study’s conducted in the USA [42]. Patients in this study also reported that positive family and social support influenced their medication intake practice which is confirmed by other studies conducted in Mexico, the Netherlands and the USA [39–41]. According to this study, self-motivation and family support were found as influential factors that allowed patients to follow their dietary advice and to stay adherent. Other studies supported these findings and stated that, as meals are usually shared in a family and eating habits are usually established within a family, adherence to dietary self-care activities depended mostly on family support [42, 43]. Patients in this study who engaged in regular walking reported being self-motivated and having positive family support, including reminders, motivation or accompaniment during walks. Studies from the past support the findings of this study reporting that lack of self-motivation and emotional support from the family hampered the regular exercise practice of the patients with chronic disease conditions [44, 45]. Since self-motivation and family support are considered as the most important factors in sustaining motivation, the creation of a support group involving both patients and their family members could be an option to share experiences and foster mutual encouragement. Health care providers should also inform and educate their patients about lifestyle changes in order to encourage their adherence. In addition, family members could be educated and counselled to support the patients [40]. Since social and family support differ across cultures and societies [39], it is also important to explore the best opportunities in different cultural and social settings.

The financial burden of the treatment of DM 2 was found as the main reason for omitting medications in this study. The same reason was also mentioned as the determining factor for non-adherence (delaying or omitting) to hospital visits (physician’s consultation and laboratory tests). The report from Ministry of Health and Family Welfare (MOHFW), Bangladesh reported that the almost non-existent public health insurance system as well as the huge OOP made patients unable to afford health care services in many cases [6]. In addition, other studies also confirmed that there was a significantly higher catastrophic expenditure on health (as OOP) among the patients with DM 2 than those without and the patients who cannot afford the high OOP and have no insurance coverage either they do not use the health care services or become non-adherent to the treatment [11, 46]. In this context, WHO suggested that prepayment and risk sharing through a mix of tax-based, social, and mandatory health insurance are the most efficient and equitable ways to protect patients’ finances and ensure access to NCD medications [47]. The key recommendations from WHO framework for access to essential medications (which include insulin) are: rationale use of essential medications at an affordable price, sustainable financing through national public and private funding, and a reliable health and supply system [46, 48]. Considering the increasing trend of DM and other NCDs, the government should consider adopting the WHO recommended framework.

Ramadan was mentioned as a reason for patient non-adherence to medication, dietary intake and physical exercise practice in this study. An irregular and/or altered medication intake practice was reported in this study during Ramadan due to fasting, which is confirmed by another study conducted among the Muslim patients with diabetes in Singapore [49]. This study also found that Ramadan had changed dietary practices in pattern with an increase in calories and sweet food, and a change in the frequency and timing of the meal which happened mainly in the evening and early morning. The same findings were reported by a review study: “Diabetes and Ramadan” [50]. Several patients of this study reported not fasting due to the fear of getting hypoglycemic attack, but there is no concrete evidence to corroborate this: some studies confirmed the risk of severe hypoglycemic events [50, 51], while others reported no significant increase in the incidence of hypoglycemic events during Ramadan [52]. With regard to physical exercise, patients in this study mentioned omitting or changing their exercise routines by having their physical exercise early in the morning to avoid getting hypoglycemic attack. Decreased physical exercise during Ramadan is evidenced in other studies as well, but a considerable level of physical exercise was recognized to be safe without any reported incidence of hypoglycemic events [49, 53]. Another study suggested that changed and/or irregular medication and dietary intake, as well as physical exercise, can potentially alter the metabolism of the body and results in a changed blood glucose level [49]. Therefore, a pre-Ramadan assessment with appropriate instructions from health care providers related to the dosage and timing of medications, proper dietary planning, physical exercise routine, and regular blood glucose monitoring could reduce the potential risk of getting hypoglycemic attack during Ramadan [49, 53].

Physical problems or poor health conditions, older age, and other co-morbidities were found to be the internal barriers to patient adherence to physical exercise in this study. Another study conducted in Serbia evidenced that physical problems and aging were the main barriers in this regard [54]. In addition, lack of safe public places for walking and unfavorable weather conditions were mentioned as the strong external barriers for patient adherence to physical exercise in this study. Lack of proper facilities such as parks or sport centers, unsafe neighborhoods, and extreme weather conditions were mentioned in other studies as well [44, 45]. Considering the growing burden of DM and other NCDs, the government should take the necessary steps to ensure safe public places for physical exercise.

Non-adherent patients showed more anger and irritation due to the need of taking regular medications in this study. One review study and another study conducted in Iran reported that patients’ negative attitudes towards their medication regimen and the perceived burden of the disease lowered the adherence to the medication intake practice and to the life style changes required for DM 2 [41, 55]. The study finding also revealed that lack of self-discipline, self-motivation, and negative health beliefs resulted in non-adherent behavior towards dietary recommendations, which is evidenced by another two studies conducted in Brazil and Botswana [43, 45].

Satisfaction with service provisions, especially the respectful behavior of the physician and the interaction and sharing of necessary information, encouraged patients interviewed to follow advice and to stay adherent. Complaints about the inattentive and unfriendly behavior of service providers, long waiting time and the cost of services were also reported. A study conducted in South Ethiopia revealed that a good patient-provider relationship has a significant influence on patient satisfaction and adherence. The study also cited that the absence of medications, cost of services and a long waiting time had a negative influence on patient satisfaction and adherence [56]. Therefore, considering the quality of service provision, it is important to improve patients´ satisfaction to increase patients’ adherence. As possible solutions, improving the appointment systems to reduce waiting time, alternative opening hours in the evenings and/or weekends, reducing the work burden of the providers through task-shifting (doctors to nurses, counselors, and other health workers) and down-referring to facilities near to home could improve patients’ satisfaction with the health care received and ensure patient adherence to long-term therapies [48].

Political instability in the country during the interview period was a challenge for this study. Despite this, most of the patients interviewed said that they were able to maintain their advised lifestyle measures and continued their lives without any huge financial consequences; which could be because we purposefully selected patients living close to the hospital). It may be the reason that this study found a contrary finding to a study conducted by Klomp et al. (2009), which reported that political instability had a negative relationship between the health care system and the health of the individuals as well as between political instability and income, which is directly linked to individual health [57].

## Conclusion and recommendation

This study provided an in-depth understanding of patients living with DM 2 and their treatment adherence (medication and other life style activities) in the context of an m-Health intervention. Positive family and social support were found to be influential factor for patient motivation and adherence. The financial burden of buying the medications, physicians’ consultations, and laboratory tests were the major barriers to treatment adherence. Lack of safe public spaces for physical exercise, unfavorable weather conditions, and political instability created some challenges for the patients to adhere to healthy lifestyle recommendations. Overall, patients had a positive perception and experience of m-Health services with regard to the management of DM 2. The results of the qualitative study confirmed our quantitative findings and suggested that m-Health could be introduced into the public health care system to reach the wider population. But several other aspects need to be considered to really increase adherence and improve disease outcomes such as financial aspects, the availability of indoor and outdoor sport centers or parks and a better integration of family members so that they can act as efficient support for DM 2 patients.

## Supplementary information

**Additional file 1.** Interview guideline for in-depth interview (intervention group).

**Additional file 2.** Interview guideline for in-depth interview (control group).

**Additional file 3.** Informed consent form for in-depth interview.

**Additional file 4.** Ethical approval from the Ethic Commission of the Medical Faculty of the University of Heidelberg, Germany.

**Additional file 5.** Ethical approval from the Ethic Commission of the Bangladesh Diabetes Association, Dhaka, Bangladesh.

## Data Availability

Data will be available on request. The first author is responsible person to contact for data access.

## Abbreviations

BADAS: Bangladesh Diabetes Association
BIHS: Bangladesh Institute of Health Sciences
BTRC: Bangladesh Telecommunication Regulatory Commission
BUHS: Bangladesh University of Health Sciences
DM 2: Diabetes Type 2
DM: Diabetes Mellitus
GLC: Graduation level completed
HRQoL: Health related quality of life
IDF: International Diabetes Federation
LMIC: Low- and middle-income country
m-Health: Mobile health
MOHFW: Ministry of Health and Family Welfare
NCD: Non-communicable diseases
NFS: No formal schooling
NGO: Non-government organization
NIPORT: National Institute of Population Research and Training
OOP: Out-of-pocket payment
PGC: Post-graduation completed
PPP: Public-private partnership
PSC: Primary school completed
SES: Socio-economic status
SMS: Short message service
SSC: Secondary school completed
TRCL: Telemedicine Reference Center Ltd.
WHO: World Health Organization
