# Supplementary material for: Understanding patients’ experience living with diabetes type 2 and effective disease management: a qualitative study following a mobile health intervention in Bangladesh
Source: BMC Health Serv Res. 2020 Jan 9;20:29. doi: 10.1186/s12913-019-4811-9 (PMC6953219; doi:10.1186/s12913-019-4811-9)
Supplement: Supplementary file 1 — Additional file 1. Interview guideline for in-depth interview (intervention group). [file 12913_2019_4811_MOESM1_ESM.pdf]

Interview guide for the in-depth interview with the intervention group of patients

UniversitätsKlinikum Heidelberg

BUHS

In-depth interview of the patients from intervention group: It is a retrospective look back into the intervention group of m-Health project for the diabetic patients for the contributing factors to the outcome of the results.

<u>Objectives:</u>

- To explore patients' view on m-Health and the process of intervention.
- To explore detail information about adherence to diet, drug, exercise and other life style changes.
- To explore the factors (personal, familial, social, economic, political) influencing adherence.

Respondent medical ID:	Interview ID:
Name of patient:	
Age of the patient:	
Address:	
Contact Mobile number:	
Date of the interview:	
Start time:	End time:

**Greetings and Introduction**<br>This part of the study is to learn about the view of patients about his/her life with diabetes and the personal coping mechanism to deal with DM. We also would like to know if your opinion about m-health intervention if you have heard about it.
Life with DM
• How would you describe your health/life with DM? Please explain

- Have you changed your personal, familial and social activities (cultural, physical, social, religious activities) due to DM? Please explain

- Are you satisfied with your life today? Please explain (Visual Scale)
  - Do you think DM affects your standard of living (familial, social, economic, physical)?

m-Health Intervention

- You were part of the group which received the m Health intervention. Can you tell us a bit more about the intervention? Which type of support did you receive? Which form? From whom?
- What did you particularly like/ not like?
- Was it helpful for you?
  - Can you tell more
- Do you think it helped you changing your knowledge, attitude, behaviour?
  - drug, diet, exercise and/or other life style changes activities?
  - How did it help you? For what?
- What do you think should be done differently in the m-Health intervention for better outcome?
- Would you be willing to pay for getting this service?
  - How much per month/Year?

Management of DM

- Do you have any difficulties to manage your diabetes?
  - Which type of difficulties?

Following medicine advices:

- Are you facing any problem following doctor's advice for drug? Please explain
  - Which type of difficulties (number of medicine, dose, duration, forgetfulness, cost)?
- How have you and your family changed daily routine to follow it? Please explain.

Doctor's consultation:

- Are you facing any problem doing your regular consultation and laboratory tests?
  - Have you ever skip any of it? If yes, please explain why?
- How have you and your family changed daily routine to follow it? Please explain.

Following Dietary Advices:

- Are you facing any problem following doctor's advice for diet?

- Which type of difficulties (not availability of advised food at home, separate menu from other family members, work outside home)?

- How have you and your family changed daily routine to follow it? Please explain.

Following physical exercise:

- Are you facing any problem following doctor's advice for physical exercise?
  - Which type of difficulties?
  - Is there anything that makes your daily physical exercise routine easier to continue?
  - Is there anything that makes your daily physical exercise routine difficult to continue?
- How have you and your family changed daily routine to follow it? Please explain.

Perception About Services

- Are you satisfied with the services available in BUHS hospital?
  - What do you particularly like?
  - What do you particularly don't like?
- Do you have any suggestion for the hospital for better service delivery?

Country Political Situation

- Do you think the unstable political condition has any impact on your daily life style and management of your diabetes?
  - Hospital visit, medicine, diet, exercise
  - Overall economic condition at personal as well as family level
- How did you cope with it? Please explain.

Closing Key Components

Is there anything you would like to add?

I will be analyzing the information and submitting a draft report to the organization within three months. I will be happy to send you a copy to review at that time, if you are interested. Thank you for your time.
