# Supplementary material for: Understanding patients’ experience living with diabetes type 2 and effective disease management: a qualitative study following a mobile health intervention in Bangladesh
Source: BMC Health Serv Res. 2020 Jan 9;20:29. doi: 10.1186/s12913-019-4811-9 (PMC6953219; doi:10.1186/s12913-019-4811-9)
Supplement: Supplementary file 2 — Additional file 2. Interview guideline for in-depth interview (control group). [file 12913_2019_4811_MOESM2_ESM.pdf]

Interview guide for the in-depth interview with the Control group of patients

In-depth interview of the patients from control group: It is a retrospective look back into the control group of m-Health project for the diabetic patients for the contributing factors to the outcome of the results.

Objectives:

- To explore patients' view on m-Health intervention.
- To explore the detail information about adherence to diet, drug, exercise and other life style changes.
- To explore the factors (personal, familial, social, economic, political) influencing adherence.

Respondent medical ID:	Interview ID:
Name of patient:	
Age of the patient:	
Address:	
Contact Mobile number:	
Date of the interview:	
Start time:	End time:

**Greetings and Introduction**<br>This part of the study is to learn about the view of patients about m-Health intervention, process and what could be improved for better outcome. We also would like to know about your life with diabetes and your personal coping mechanism to deal with DM.
Life with DM
• How would you describe your health/life with DM? Please explain

- Have you changed your personal, familial and social activities (cultural, physical, social, religious activities) due to DM? Please explain

- Are you satisfied with your life today? Please explain (Visual Scale)
  - Do you think DM affects your standard of living (familial, social, economic, physical)?

m-Health Intervention

- Have you heard about the m-Health intervention that is taking place at BUHS? From where? Can you explain us what you heard about it?

If the person is not able to explain, or not correctly explaining, please explain by yourself at first in a few words.

- Do you think it could be helpful for you?
- Do you think it could help you changing your behaviour?
  - drug, diet, exercise and/or other life style changes activities?
  - What do you that how will it help you? For what?
- Do you have any suggestion for improving the m-Health intervention for better outcome if you already know about it?
- Are you willing to pay for getting this service?
  - How much per month/Year?

Management of DM

- Do you have any difficulties to manage your diabetes?
  - Which type of difficulties?

Following medicine advices:

- Are you facing any problem following doctor's advice for drug? Please explain
  - Which type of difficulties (number of medicine, dose, duration, forgetfulness, cost)?
- How have you and your family changed daily routine to follow it? Please explain.

Doctor's consultation:

- Are you facing any problem doing your regular consultation and laboratory tests?
  - Have you ever skip any of it? If yes, please explain why?
- How have you and your family changed daily routine to follow it? Please explain.

Following Dietary Advices:

- Are you facing any problem following doctor's advice for diet?

- Which type of difficulties (not availability of advised food at home, separate menu from other family members, work outside home)?
- How have you and your family changed daily routine to follow it? Please explain.

Following physical exercise:

- Are you facing any problem following doctor's advice for physical exercise?
  - Which type of difficulties?
  - Is there anything that makes your daily physical exercise routine easier to continue?
  - Is there anything that makes your daily physical exercise routine difficult to continue?
- How have you and your family changed daily routine to follow it? Please explain

Perception About Services

- Are you satisfied with the services available in BUHS hospital?
  - What do you particularly like?
  - What do you particularly don't like?
- Do you have any suggestion for the hospital for better service delivery?

Country Political Situation

- Do you think the unstable political condition has any impact on your daily life style and management of your diabetes?
  - Hospital visit, medicine, diet, exercise
  - Overall economic condition at personal as well as family level
- How did you cope with it? Please explain.

Closing Key Components

Is there anything you would like to add?

I will be analysing the information and submitting a draft report to the organization within three months. I will be happy to send you a copy to review at that time, if you are interested.

Thank you for your time.
