# Supplementary material for: Understanding patients’ experience living with diabetes type 2 and effective disease management: a qualitative study following a mobile health intervention in Bangladesh
Source: BMC Health Serv Res. 2020 Jan 9;20:29. doi: 10.1186/s12913-019-4811-9 (PMC6953219; doi:10.1186/s12913-019-4811-9)
Supplement: Supplementary file 3 — Additional file 3. Informed consent form for in-depth interview. [file 12913_2019_4811_MOESM3_ESM.pdf]

Informed consent form for in-depth interview

I want to thank you for giving me time to talk to you today.

My name is ______________________________________ and I would like to talk to you about your experiences living with diabetes. The answers will be used only for research purpose and maintained fully confidential.

I would be pleased if you participated in the interview, but your participation is absolute **VOLUNTARY**. The interview will take about an hour. There will be **no** personal benefits you gain if you do participate. If you would like to participate, you are not obliged to answer **all** questions. If any question seems too difficult/sensitive to you, you just say that ***you don't want to answer***.

If you don't want to participate, please feel free to say **no**. I will respect your decision. If you ***do not want to participate*** or if ***you choose to end the interview*** at any point of time, there will be **no** need to give any explanation and **no** negative consequences will happen afterwards regarding your medical services here. If you choose to terminate the interview, all your information will be discarded if you want. However, you also could decide give us consent to continue use your data for study purpose.

The interview will take around one hour. I will record the full session as well as notes. I need to record the session as I cannot be so fast in writing following your talk and I don't want to miss any of your valuable comments. As we are on tape, please be sure to speak up loud enough so that we don't miss any of your comments.

The information will be written down and recorded and therefore evaluated and used for writing scientific paper that goes to the "University Hospital Heidelberg" in Germany and "Bangladesh University of Health Sciences" in Bangladesh. The data will be secured from unauthorized access and passed on anonymously.

Do you have any questions to that?

For further questions or concerns, **please contact**:

Farzana Yasmin (Doctoral Student, Institute of Public Health, University of Heidelberg, Germany)

Tel: +49 176 84776531, Email: farzana@stud.uni-heidelberg.de

Are you willing to participate in this interview?

Signature of interviewee: ___________________

Signature of interviewer: ____________________

Date of the signature: _______/_______/_________ (dd/mm/yyyy)
