# Supplementary material for: Understanding patients’ experience living with diabetes type 2 and effective disease management: a qualitative study following a mobile health intervention in Bangladesh
Source: BMC Health Serv Res. 2020 Jan 9;20:29. doi: 10.1186/s12913-019-4811-9 (PMC6953219; doi:10.1186/s12913-019-4811-9)
Supplement: Supplementary file 4 — Additional file 4. Ethical approval from the Ethic Commission of the Medical Faculty of the University of Heidelberg, Germany. [file 12913_2019_4811_MOESM4_ESM.pdf]

Medizinische Fakultät Heidelberg

Ethikkommission
Medizinische Fakultät Heidelberg

Ethikkommission der Med. Fak. HD | Alte Glockengießerei 11/1 | D-69115 Heidelberg

Herrn Prof. Dr. med. Rainer Sauerborn
Institute of Public Health
Universitätsklinikum Heidelberg
Im Neuenheimer Feld 324
69120 Heidelberg

Alte Glockengießerei 11/1
D-69115 Heidelberg

☏ +49 6221 33822 0 (Empfang)
+49 6221 3382222
ethikkommission-I@med.uni-heidelberg.de

www.medizinische-fakultaet-hd.uni-heidelberg.de/ethikkommission

Vorsitz:
Prof. Dr. med. Thomas Strowitzki

Stellv. Vorsitz:
Prof. Dr. med. Johannes Schröder
Prof. Dr. med. Klaus Herfarth

Geschäftsleitung:
Dr. med. Verena Pfeilschifter

Sonstige Studien:

Sina Bittar, M.A.
☏ +49 6221 3382218
+49 6221 3382222
Sina.Bittar@med.uni-heidelberg.de

Dr. rer. nat. Marion Teichmann
☏ +49 6221 3382219
+49 6221 3382222
Marion.Teichmann@med.uni-heidelberg.de

24.09.2013
ts-sb

Berufsrechtliche Beratung

Unser Zeichen: S-281/2013 (Bitte stets angeben)

Titel: **M-health Influence Patient Adherence and Allows Cost Effective Management of Type 2 Diabetes in Bangladesh**

Eingereichte Unterlagen:
<u>Ersteinreichung vom 24.06.2013:</u>
Anschreiben vom 28.05.2012
Summary
Checkliste Sonstige Studien
Formular für Erstantrag vom 28.05.2013
Information Form Version 1.0 vom 28.05.2013
Consent Form Version 1 vom 28.05.2013
Study Protocol Version 1 vom 28.05.2013
CV Prof. Dr. Rainer Sauerborn vom 28.05.2013
Interview Questionnaire Form Version 1
<u>Nachreichung vom 07.07.2013:</u>
Anschreiben
Summary
Information Form Version 1.0 vom 28.05.2013
Consent Form key informant Version 2 vom 09.07.2013
Consent Form Patient Version 2 vom 09.07.2013
Study Protocol Version 2 vom 09.07.2013

Sehr geehrter Herr Professor Sauerborn,

die Ethikkommission hat Ihr Forschungsvorhaben in der Sitzung am 22.07.2013 beraten und hat **keine Bedenken gegen die Studie.**

Sie gibt jedoch folgende Empfehlungen bzw. Hinweise:

<u>Allgemein:</u>

1. Für Familienangehörige ist eine separate Informationsschrift und Einwilligungserklärung zu vorzusehen.

<u>Study Protocol:</u>

2. Das biometrische Konzept der Studie sollte bzgl. der nachfolgenden Punkte ergänzt werden:
3. Die geplanten Fallzahlen sollten für die verschiedenen Gruppen angegeben und soweit als möglich begründet werden.

ISO 9001:2008
399893 QM

Bankverbindung:
Baden-Württembergische Bank Stuttgart
Konto-Nr.: 7421 500 429
BLZ: 600 501 01
SWIFT/BIC Code: SOLADEST
IBAN-Nr.: DE 64600501017421500429

4. Die Ziel- und Einflussgrößen (u.U. unterteilt in primäre, sekundäre und von Survey-Daten abgeleitete) sollten (u.U. bezugnehmend auf die verschiedenen Messinstrumente) in einem eigenen Abschnitt des Protokolls beschrieben werden.
5. Die für die Auswertung vorgesehenen statistischen Verfahren und die Auswertungsstrategie sollten, z.B. in Kapitel 26, zumindest so detailliert und zielgerichtet dargestellt werden, dass erkennbar ist, wie die Fragestellungen der Studie evaluiert werden sollen. Dazu sollte der in Kapitel 18 beschriebene Difference-in-Difference Ansatz für die primären Zielkriterien detailliert und angewendet und auch die „ x-Variablen“ spezifiziert werden, mit denen der Propensityscore bestimmt werden soll. Eine biometrische Beratung wird empfohlen mit entsprechender Ergänzung in Kapitel 05, Seite 1.
6. Kapitel 25 „Randomization Method“ sollte als non-applicable“ deklariert werden.
7. Die Kapitelnummerierung sollte überarbeitet werden.
8. Das aktuelle Studienprotokoll ist vom Studienleiter zu unterschreiben.

Information sheet:

9. Der Inhalt der Fragen sollte kurz umrissen werden.

Consent form key informant patient/family member:

10. Es sollte ergänzend ein Hinweis eingefügt werden, aus dem hervorgeht, dass die Probanden der Teilnahme an der Studie freiwillig zustimmen.
11. Wenn nur Daten des Patienten erhoben werden, sollte die Formulierung „mydata“ bzgl. der keyinformants und Familienangehörigen korrigiert werden.
12. Die Daten können nur anonymisiert oder pseudonymisiert weitergegeben werden – die Angaben unterscheiden sich in der Informationsschrift und in der Einwilligung und sollten übereinstimmen.

Consent form patient/family member:

13. Es sollte ein Absatz eingefügt werden, aus dem hervorgeht, dass die Patienten/Probanden der Teilnahme an der Studie freiwillig zustimmen.

Study Protocol:

14. Laut telefonischer Auskunft von Dr. Souares werden alle Daten pseudonymisiert. Kapitel 43 „Data Protection“ sollte entsprechend überarbeitet werden: „….Personal data will only be passed on anonymously or under pseudonym.”

Wir wünschen Ihnen bei der Durchführung der Studie viel Erfolg.

Bitte leiten Sie das Ergebnis der berufsrechtlichen Beratung und die studienrelevante Korrespondenz allen teilnehmenden Ärzten in unserem Zuständigkeitsbereich weiter.

Mit freundlichen Grüßen

Digital unterschrieben von Dr. Thomas Strowitzki:PN
DN: cn=Dr. Thomas Strowitzki:PN, pseudonym=Dr. Thomas Strowitzki:PN, serialNumber=0010000000011954230002
Datum: 2013.09.24 14:28:02 +02'00'

Prof. Dr. med. Thomas Strowitzki
Vorsitzender

©EKHD-ZB-S-Dok-Nr-DV-070-130514

Allgemeine Hinweise:

- Änderungen in Organisation und Ablauf der Studie sind der Kommission, zusammen mit einer Bewertung der Nutzen-Risiko-Relation, umgehend mitzuteilen. Sowohl die **Antragsnummer** als auch die **geänderten Passagen** sollten in den betreffenden Unterlagen **deutlich gekennzeichnet** sein, da anderenfalls keine zügige Bearbeitung möglich ist.
- Die Ethikkommission der Medizinischen Fakultät Heidelberg arbeitet gemäß den nationalen gesetzlichen Bestimmungen und den ICH-GCP-Richtlinien. Ihren Beratungen liegt die Deklaration des Weltärztebundes von Helsinki in der jeweils aktuellen Fassung zugrunde.
- Unabhängig vom Beratungsergebnis macht die Ethikkommission Sie darauf aufmerksam, dass die ethische und rechtliche Verantwortung für die Durchführung einer Studie beim Leiter der Studie und bei allen teilnehmenden Ärzten liegt.

©EKHD-ZB-S-Dok-Nr-DV-070-130514
