# Supplementary material for: Understanding patients’ experience living with diabetes type 2 and effective disease management: a qualitative study following a mobile health intervention in Bangladesh
Source: BMC Health Serv Res. 2020 Jan 9;20:29. doi: 10.1186/s12913-019-4811-9 (PMC6953219; doi:10.1186/s12913-019-4811-9)
Supplement: Supplementary file 5 — Additional file 5. Ethical approval from the Ethic Commission of the Bangladesh Diabetes Association, Dhaka, Bangladesh. [file 12913_2019_4811_MOESM5_ESM.pdf]

বাংলাদেশ ডায়াবেটিক সমিতি
DIABETIC ASSOCIATION OF BANGLADESH

Memo No. BADAS-ERC/EC/13/00110 Date: October 27, 2013

To

Farzana Yasmin
Doctoral student from Institute of Public Health
Heidelberg University
Germany

Subject: Ethical Clearance

The Ethical Review Committee (ERC) of the Diabetic Association of Bangladesh (BADAS) has approved your protocol on "Does mobile-health influence patient adherence and allows cost effective management of Type-2 diabetes in Bangladesh?"

K. M. S. Aziz
(Dr. KMS Aziz)
Chairman
Ethical Review Committee

বাংলাদেশ ডায়াবেটিক সমিতি
DIABETIC ASSOCIATION OF BANGLADESH

Memo No. BADAS-ERC/EC/13/00110 Date: October 27, 2013

To

Farzana Yasmin
Doctoral student from Institute of Public Health
Heidelberg University
Germany

Subject: Ethical Clearance

The Ethical Review Committee (ERC) of the Diabetic Association of Bangladesh (BADAS) has approved your protocol on "Does mobile-health influence patient adherence and allows cost effective management of Type-2 diabetes in Bangladesh?"

K. M. S. Aziz
(Dr. KMS Aziz)
Chairman
Ethical Review Committee

122 KAZI NAZRUL ISLAM AVENUE SHAHBAG DHAKA 1000 BANGLADESH PHONE : 8616641-50, 880-2-9661551 FAX : 9677772, Web : www.dab-bd.org
